# Prevalence and genotypic characterization of zoonotic intestinal protozoan parasites in transboundary migratory Mongolian Gazelles (*Procapra gutturosa*): the first survey

**DOI:** 10.1017/S0031182024000854

**Published:** 2024-09

**Authors:** Sichao Gao, Bin Hu, Gaojian Li, Xing An, Yanan Xing, Shuyi Han, Yan Chun, Lingling Han, Du Xi, Wenchao Li, Hongxuan He

**Affiliations:** 1 Anhui Science and Technology University, Fengyang 233100, China; 2 Institute of Zoology, Chinese Academy of Sciences, Beijing 100101, China; 3 University of Chinese Academy of Sciences, Beijing 100049, China; 4 Inner Mongolia Minzu University, Tongliao 028000, China; 5 Center for Disease Control and Prevention, Xinbarhu Right Banner, Inner Mongolia Autonomous Region, Hulunbuir City 021300, China

**Keywords:** *Blastocystis* sp, *Cyclospora cayetanensis*, *Cystoisospora belli*, *Cryptosporidium* spp, host-adapted genotypes, Mongolian gazelle, zoonotic intestinal protozoan

## Abstract

The migration of Mongolian gazelles (*Procapra gutturosa*) poses a potential risk of outbreak for zoonotic intestinal protozoan parasite infections. This study aims to investigate the infection status of zoonotic intestinal protozoan parasites in these migratory Mongolian gazelles. We collected 120 fecal samples from Mongolian gazelles during their migration from Mongolia to China in December 2023. These samples were analysed using amplification and sequencing of partial SSU rRNA genes to detect the 4 presence of zoonotic intestinal protozoan parasites and characterize their genotypes. Our analysis revealed the presence of several zoonotic intestinal protozoan parasites in the sampled Mongolian gazelles. *Cryptosporidium* spp. was detected in 14.17% (17/120) of the samples, followed by *Cystoisospora belli* in 13.33% (16/120), *Blastocystis* sp. in 16.67% (20/120) and *Cyclospora cayetanensis* in 30.00% (36/120). Moreover, we identified novel host-adapted genotypes of *Cryptosporidium* spp. and *C. belli*, as well as the presence of ST2 and ST13 *Blastocystis* sp. subtypes, while distinct genotypes were found in *Blastocystis* sp. and *C. cayetanensis*. This study revealed the status of 4 prevalent zoonotic intestinal protozoan parasite infections in Mongolian gazelles and provided crucial insights into their characteristics. The prevalence of these parasites in the population highlights the potential risk of cross-border transmission of infectious diseases associated with long-distance migration. Furthermore, the identification of novel genotypes contributes to our understanding of the genetic diversity and adaptation of these parasites. These findings can inform the development of protective measures to mitigate the impact of these infections on the health and survival of Mongolian gazelles.

## Graphical abstract

**Figure:**
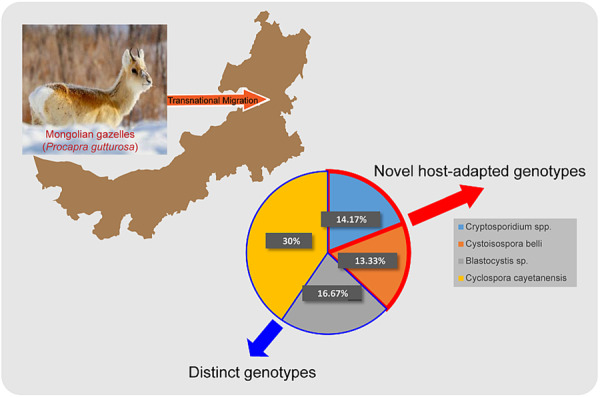

## Introduction

Mongolian gazelles (*Procapra gutturosa*), a species native to the temperate grasslands of Eurasia, are important herbivores. In the 1970s, there were over 3 million wild Mongolian gazelles in the grasslands of Inner Mongolia, China. However, the population has declined to approximately 1000 individuals today (Ito *et al*., 2006; Shi *et al*., 2023). Based on the rate and extent of population decline, Mongolian gazelles have been classified as a National First-class Protected Animal in China (Jiang *et al*., 2021). Mongolian gazelles are primarily distributed in the eastern grasslands of Mongolia and in Hulunbuir, Xilingol and Ulanqab in China, with a concentrated presence in the border regions between the 2 countries. They also have extensive migratory movements, typically undergoing large-scale migrations in spring and autumn. During winter, a significant number of Mongolian gazelles cross the border into China for wintering before returning to Mongolia (Bi *et al*., 1997). To facilitate the free migration of Mongolian gazelles and other wildlife, China has established multiple approximately wildlife corridors on the frequently traversed border fences, considering the living habits of Mongolian gazelles and the topography and water distribution in protected areas. However, the regional movements and long-distance migrations of wildlife can significantly increase the cross-border transmission risks of infectious diseases, particularly in the context of global climate change and urbanization. The distribution areas, movement patterns and migration routes of wildlife may undergo corresponding changes, leading to accelerated disease outbreaks, especially in border regions. Furthermore, due to Mongolia being an endemic area for foot-and-mouth disease (McFadden *et al*., 2015), and peste des petits ruminants (Benfield *et al*., 2021), the risk of disease transmission into China through infected Mongolian gazelles poses an increasing threat to public health security.

Zoonotic intestinal protozoan parasites primarily spread through waterborne or soil-transmitted, with human infections typically originating from a variety of domestic and wild animals. Consequently, these diseases pose significant public health challenges to both humans and wildlife, especially in poor and developing countries. (Belete *et al*., 2021; Maganga *et al*., 2023). Four zoonotic protozoan intestinal parasites-*Cryptosporidium* spp*., Cystoisospora belli, Blastocystis* sp. and *Cyclospora cayetanensis,* are widespread threats to human health worldwide. Among these, *Cryptosporidium* spp. is a widespread zoonotic parasite known to cause global outbreaks of waterborne and foodborne gastroenteritis. Human infection with *Cryptosporidium* spp. generally does not display obvious clinical symptoms, but severe cases can lead to changes in coat colour, loss of appetite, wasting, diarrhoea and even death (Cacciò and Chalmers, 2016; Zahedi and Ryan, 2020). One notable characteristic of *Cryptosporidium* is its extensive genetic variation, resulting in the existence of more than 38 species and over 60 genotypes of this parasite (Feng *et al*., 2018). *Cystoisospora belli*, previously known as *Isospora belli*, is a parasite responsible for causing an intestinal disease called cystoisosporiasis. This protozoan parasite is opportunistic and often affects immunocompromised individuals, particularly those with HIV infection or living in institutional settings (Legua and Seas, 2013; Rial-Crestelo *et al*., 2022). *Blastocystis* sp. is an anaerobic single-celled intestinal parasite that can infect both animals and humans (Deng *et al*., 2019). *Blastocystis* has been found to exist in approximately 1 to 2 billion people worldwide, making it highly prevalent (Tan, 2008). Apart from humans, this parasite is frequently detected in various animal hosts, including non-human primates and other mammals such as ungulates, perissodactyls, proboscideans, rodents and marsupials, as well as birds, reptiles, amphibians, fish, annelids and insects (Wang *et al*., 2018). *Cyclospora* sp. is an emerging pathogen responsible for global outbreaks of cyclosporiasis, posing a threat to the health of both humans and various animal species. Since its discovery, numerous studies have reported the molecular characteristics and zoonotic potential of Cyclospora in various animals (Ortega and Sanchez, 2010). On the other hand, *C. cayetanensis* has been confirmed as a zoonotic pathogen and is the only known genotype capable of infecting humans. Currently, documented hosts of Cyclospora include dogs, cattle, chickens, non-human primates and some rodents (Totton *et al*., 2021).

Transboundary migratory animals can serve as vectors for disease transmission, particularly when they come into close contact with humans or other animals. Mongolian gazelles are important wild animal resources, and therefore, it is necessary to further study the relationship between migratory Mongolian gazelles and diseases in order to develop effective conservation measures and reduce the risk of disease transmission. Therefore, this study aims to investigate the prevalence of common zoonotic intestinal protozoan parasites, including *Cryptosporidium* spp., *C. belli*, *Blastocystis* sp., and *C. cayetanensis*, in a large-scale population of Mongolian gazelles that migrated across the border and entered China from Mongolia in early December 2023. By studying the infection rate of those protozoan in Mongolian gazelles, the risk of zoonotic diseases can be assessed, and appropriate preventive measures can be implemented to reduce the occurrence and spread of outbreaks. This research holds immense importance for wildlife conservation, zoonotic disease prevention and control, as well as public health.

## Methods and methods

### Ethical statement

This study was conducted in strict accordance with the Guidelines for the Care and Use of Animals in Research published by the Institute of Zoology, Chinese Academy of Sciences.

### Collection of stool samples

A population of Mongolian gazelles (approximately 4500–6000) migrating from the Sino-Mongolian border into China was selected as the target for monitoring in this study. From December 12th to 14th, 2023, within the activity area of the wild Mongolian gazelle population in the Hinggan League of Inner Mongolia, China (North Latitude N: 46°41′48.43″ East Longitude E: 119°58′30.60″), investigations were conducted by randomly sampling one specimen from each plot of land (each with an area of 20 m × 20 m) to ensure the representativeness of the sample and the accuracy of the conclusions drawn for the entire population. The collected fecal samples were immediately transported to the laboratory at the Institute of Zoology, Chinese Academy of Sciences in Beijing using dry ice and stored at −80°C for further experimentation.

### Stool sample collection and DNA extraction

Total genomic DNA was extracted from 200 mg Stool according to the instruction of E.Z.N.A. ® Stool DNA Kit Stool Whole Genome Extraction Kit (OMEGA Biotek INCD4015-01, USA). The extracted Stool DNA was stored in a refrigerator at −20°C for further PCR assays.

### PCR amplification

Nested PCR was employed to detect the small subunit ribosomal ribonucleic acid (SSU rRNA) gene of *Cryptosporidium* spp., *C. belli*, *Blastocystis* sp., and *C. cayetanensis*. The SSU rRNA gene of *Cryptosporidium* spp. (Xiao *et al*., 2001), *C. belli* (Zhang, 2022), *Blastocystis* sp. (Wang *et al*., 2013) and *C. cayetanensis* (Li *et al*., 2011) were individually examined using nested PCR following established protocols. Detailed primer information and PCR procedures can be found in Table 1. Each amplification reaction included both positive and negative controls. The amplified DNA fragments underwent electrophoresis on a 1.5% agarose gel with Gel-Red. Subsequently, the resulting electrophoresis patterns were visualized, analysed using a gel imaging system, and documented through photographs.

**Table 1.** Forward and reverse primers used for the detection of pathogenic intestinal protozoan parasites in *Procapra gutturosa*

Gene	Primers			Amplication protocol	Size	Ref
*Cryptosporidium* spp. SSU gene	1st	Forward	5’-TTCTAGAGCTAATACATGCG-3’	94°C: 3 min, 35 cycles: (94°C: 45 s, 55°C: 45 s, 72°C:1 min) 72°C: 7 min		Xiao *et al*. (2001)
	PCR	Reverse	5’-CCCATTTCCTTCGAAACAGGA-3’			
	2nd	Forward	5’-GGAAGGGTTGTATTTATTAGATAAAG-3’	94°C: 3 min, 35 cycles: (94°C: 45 s, 55°C: 45 s, 72°C:1 min) 72°C: 7 min	864 bp	
	PCR	Reverse	5’-AAGGAGTAAGGAACAACCTCCA-3’			
*Cystoisospora belli* SSU gene	1st	Forward	5’-TGTTTATTAGATACAGAACCAACCCAC-3’	94°C: 5 min, 35 cycles: (94°C: 45 s, 55°C: 45 s, 72°C: 1 min) 72°C: 10 min		Zhang (2022)
	PCR	Reverse	5’-CTTTCTATGTCTGGACCTGGTGAG-3’			
	2nd	Forward	5’-TAACCGAACGGATCGCGTTAT-3’	94°C: 5 min, 35 cycles: (94°C: 45 s, 52°C: 45 s, 72°C: 1 min) 72°C: 10 min	530 bp	
	PCR	Reverse	5’-TAACCGAACGGATCGCGTTAT-3’			
*Blastocystis* sp. SSU gene	1st	Forward	5’-GGGATCCTGATCCTTCCGCAGGTTCACCTAC-3’	94°C: 5 min, 40 cycles: (95°C: 30 s, 65°C: 45 s, 72°C: 1 min) 72°C: 10 min		Wang *et al*. (2013)
	PCR	Reverse	5’-GGGATCCTGATCCTTCCGCAGGTTCACCTAC-3’			
	2nd	Forward	5’-GGAGGTAGTGGACAATAAATC-3’	94°C: 5 min, 35 cycles: (95°C: 30 s, 54°C: 45 s, 72°C: 1 min) 72°C: 10 min	1100 bp	
	PCR	Reverse	5’- CGTTCATGATGAACAATTAC -3’			
*Cyclospora* sp. SSU gene	1st	Forward	5’-AACCTGGTTGATCCTGCCAGT-3’	94°C: 5 min, 35 cycles: (95°C: 45 s, 53°C: 45 s, 72°C: 1 min) 72°C: 10 min		Li *et al*. (2011)
	PCR	Reverse	5’-AAAGTTCCGGAACACCAAC-3’			
	2nd	Forward	5’-GCTTGTCTCAAAGATTAAGCCATG-3’	94°C: 5 min, 35 cycles: (94°C: 45 s, 53°C: 45 s, 72°C: 1 min) 72°C: 10 min	680 bp	
	PCR	Reverse	5’-AAGGCTACCGGAAGAAAGCC-3’			

### Gene sequencing

The secondary PCR positive products were sequenced using Sanger sequencing method, and the resulting sequences were spliced in both directions by Shenzhen Huada Gene Co Ltd (Beijing, China). Following the comparison of sequencing results, DNAMAN software was employed to correct splicing and assemble the sequences. The successfully sequenced sequences were then searched and aligned against related sequences with higher homology on the GenBank database hosted by the National Center for Biotechnology Information (NCBI). To further validate the alignment, the downloaded sequences and sequencing results were assembled and compared using Clustal X2.1 software.

### Analysis of the evolutionary relationships

As reference genes, we selected host genotypes and environment sequences of Cryptosporidium sp., including chipmunk genotype I, muskrat genotype II, Brandt's vole, Microtus arvalis, chipmunk, tyzzeri, meleagridis, felis, andersoni, hominis, ubiquitum and other taxa. Phylogenetic tree was constructed based on SSU rRNA gene using MEGA9.0 software and the Neighbour-joining method, employing the bootstrap test with 1000 replicates and the Tamura 3-parameter model. The outgroup consisted of *Eimeria* sp. (GenBank: KT305927.1).

For *C. belli* genotypes, reference genes were selected from samples isolated from humans, *Columba livia* domestica, *Parus major*, *Turdus migratorius*, domestic canaries and other taxa. Phylogenetic tree was constructed based on SSU rRNA gene using MEGA9.0 software and the Neighbour-joining method, employing the bootstrap test with 1000 replicates and the Jukes–Cantor model. The outgroup consisted of *Giardia intestinalis* (GenBank: OR689423.1).

For *Blastocystis* sp. genotypes, reference genes were selected from genotypes including ST1, ST2, ST3, ST4, ST5, ST6, ST7, ST8, ST9, ST10, ST12, ST13 and ST14 serotypes, isolated from *Homo sapiens*, *Cervus nippon*, *Alouatta palliata aequatoriali*, *Camelus dromedarius*, *Macaca fuscata*, *Moschus berezovskii*, *Anser cygnoides*, *Wallabia bicolor mastersii*, *Gallus domesticus*, *Ruttus novercious*, *Rusa unicolor*, *Elaphodus cephalopus* and *Ovis aris*. Phylogenetic tree was constructed based on SSU rRNA gene using MEGA9.0 software and the Neighbour-joining method, employing the bootstrap test with 1000 replicates and the Jukes–Cantor model. The outgroup consisted of *Histomonas meleagridis* (GenBank: AJ920323.1).

For *C. cayetanensis* genotypes, reference genes were selected from samples isolated from *H. sapiens*, *Macaca mulatta*, Du-long cattle, dairy cattle farm, Holstein cattle, dairy cattle, *Macaca fascicularis*, baboon and other taxa. Phylogenetic tree was constructed based on SSU rRNA gene using MEGA9.0 software and the Neighbour-joining method, employing the bootstrap test with 1000 replicates and the Tamura 3-parameter model. The outgroup consisted of *Entamoeba histolytica* (GenBank: OR236732.1).

## Results

### The infection rates of Cryptosporidium spp., *C. belli*, Blastocystis sp., and *C. cayetanensis* in Mongolian gazelles

Positive samples were determined based on successful sequence alignment in the sequencing results. Among the 120 samples, the infection rates results indicated that *Cryptosporidium* spp. was detected in 14.17% (17/120), followed by *C. belli* in 13.33% (16/120), *Blastocystis* sp. in 16.67% (20/120) and *C. cayetanensis* in 30.00% (36/120) (Table 2). All PCR-positive samples from the *Cryptosporidium* SSU rRNA gene were sequenced, and 2 complete and representative sequences were submitted to NCBI GenBank (Accession Numbers: **PP159013** and **PP159016**) after excluding poor quality sequences due to sequence fragmentation, incompleteness, or inaccurate sequence content. Similarly, the sequences of the SSU rRNA gene locus of *C. belli* were submitted to GenBank, acquiring the sequence accession numbers: **PP159019** and **PP159020**. Additionally, the sequences of the SSU rRNA gene locus of *Blastocystis* sp. were sequenced and submitted to NCBI GenBank, resulting in the assignment of sequence accession numbers: **PP159017** and **PP159018**. Lastly, the sequences of the SSU rRNA gene locus of *C. cayetanensis* were sequenced and submitted to NCBI GenBank, acquiring the sequence accession numbers: **PP159021** and **PP159025**.

**Table 2.** The prevalence (No. Positive/No. Tested) of pathogenic intestinal protozoa in *Procapra gutturosa*

Pathogen type	No. positives	No. tested	Prevalence, %
*Cryptosporidium* spp.	17	120	14.17
*Cystoisospora belli*	16	120	13.33
*Blastocystis* sp.	20	120	16.67
*Cyclospora cayetanensis*	36	120	30.00

### Evolutionary relationships analysis of SSU rRNA gene in Cryptosporidium spp. isolated from Mongolian gazelles

**Figure 1.**
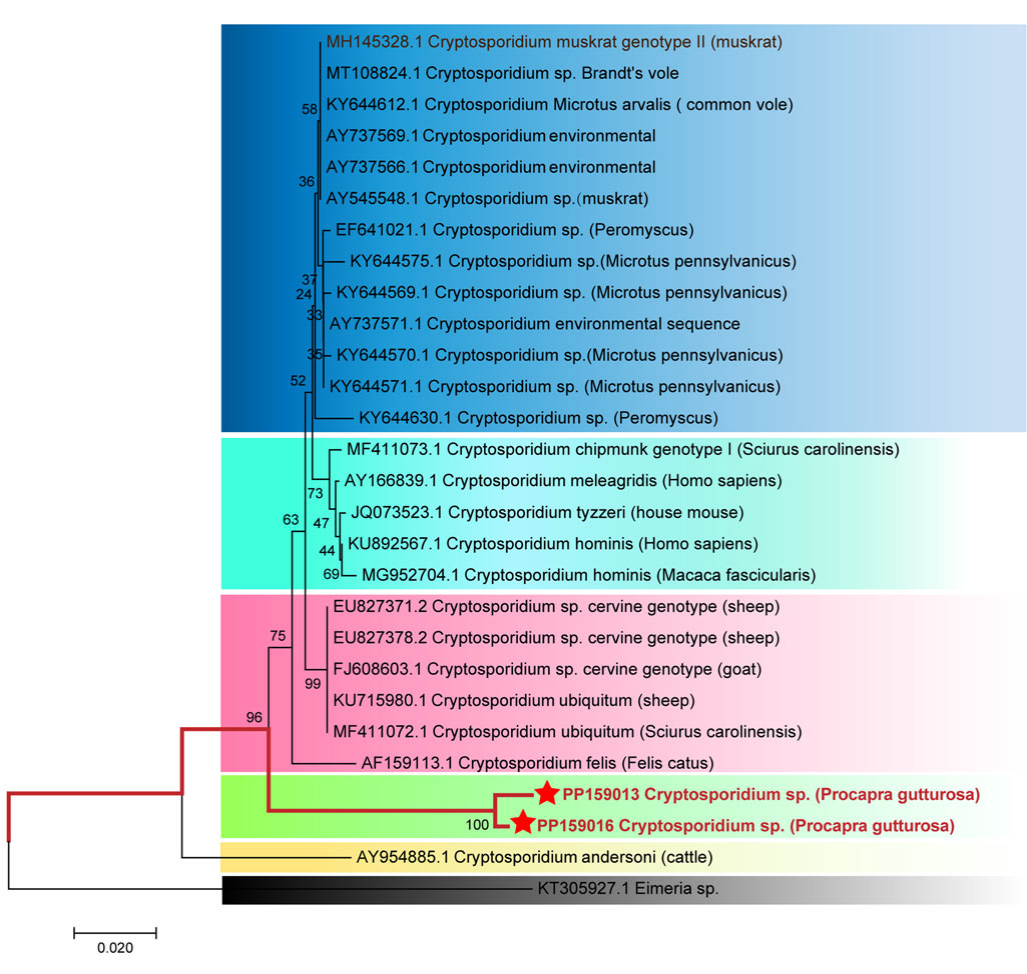
The Evolutionary relationships of *Cryptosporidium* sp. genotypes identified in the present study and other known genotypes and species on GenBank were inferred by a Neighbour-joining method of SSU rRNA gene using the bootstrap test (1000 replicates) and Tamura 3-parameter model method. The *Eimeria* sp. (GenBank: KT305927.1) were used as the outgroup. The Solid red lines and red 5-pointed stars represent novel genotypes identified in this study.

A phylogenetic tree (Fig. 1) was constructed using the SSU rRNA gene sequence of a reference *Cryptosporidium* spp. The *Cryptosporidium* spp. genotypes discovered in Mongolian gazelles (PP159013 and PP159016) formed a distinct branch (97 to 98% homology), indicating their potential as host-specific genotypes for Mongolian gazelles. Based on the molecular characteristics and host specificity differences, we designated the newly identified genotype as *Cryptosporidium Mongolian gazelle* genotype.

### Evolutionary relationships analysis of SSU rRNA gene in *C. belli* isolated from Mongolian gazelles

**Figure 2.**
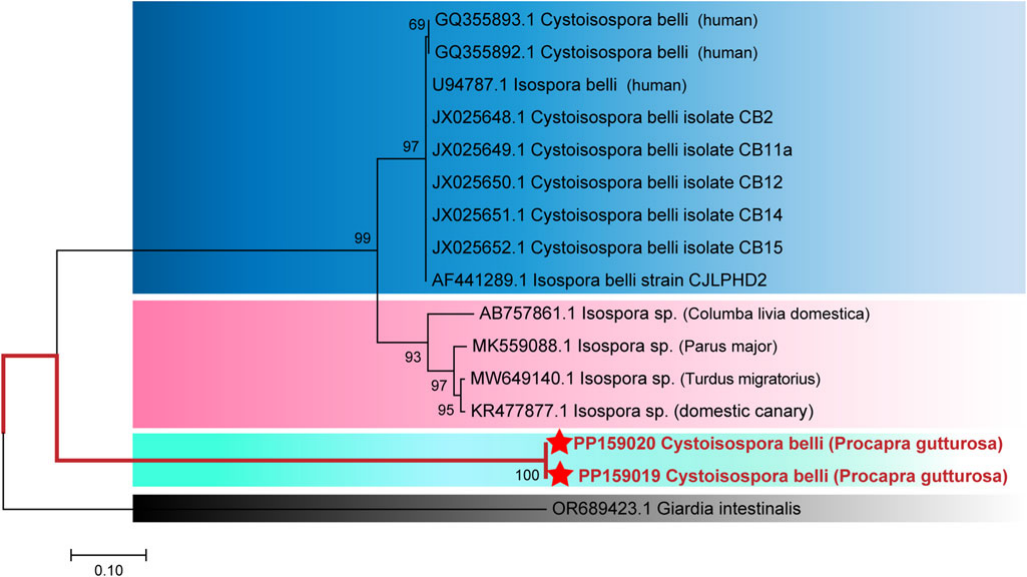
The Evolutionary relationships of *Cystoisospora belli* genotypes identified in the present study and other known genotypes and species on GenBank were inferred by a Neighbour-joining method of SSU rRNA gene using the bootstrap test (1000 replicates) and Jukes–Cantor model method. The *Giardia intestinalis* (GenBank: OR689423.1) were used as the outgroup. The Solid red lines and red 5-pointed stars represent novel genotypes identified in this study.

A phylogenetic tree (Fig. 2) was constructed using the SSU rRNA gene sequence of a reference *C. belli*. The *C. belli* genotypes found in Mongolian gazelles (PP159019 and PP159020), which formed a distinct branch, had a homology of 99%, suggesting their potential as host-specific genotypes for Mongolian gazelles. Considering the molecular characteristics and differences in host specificity, we designated the newly identified genotype as *C. belli* Mongolian gazelle genotype.

### Evolutionary relationships analysis of SSU rRNA gene in Blastocystis sp. isolated from Mongolian gazelles

**Figure 3.**
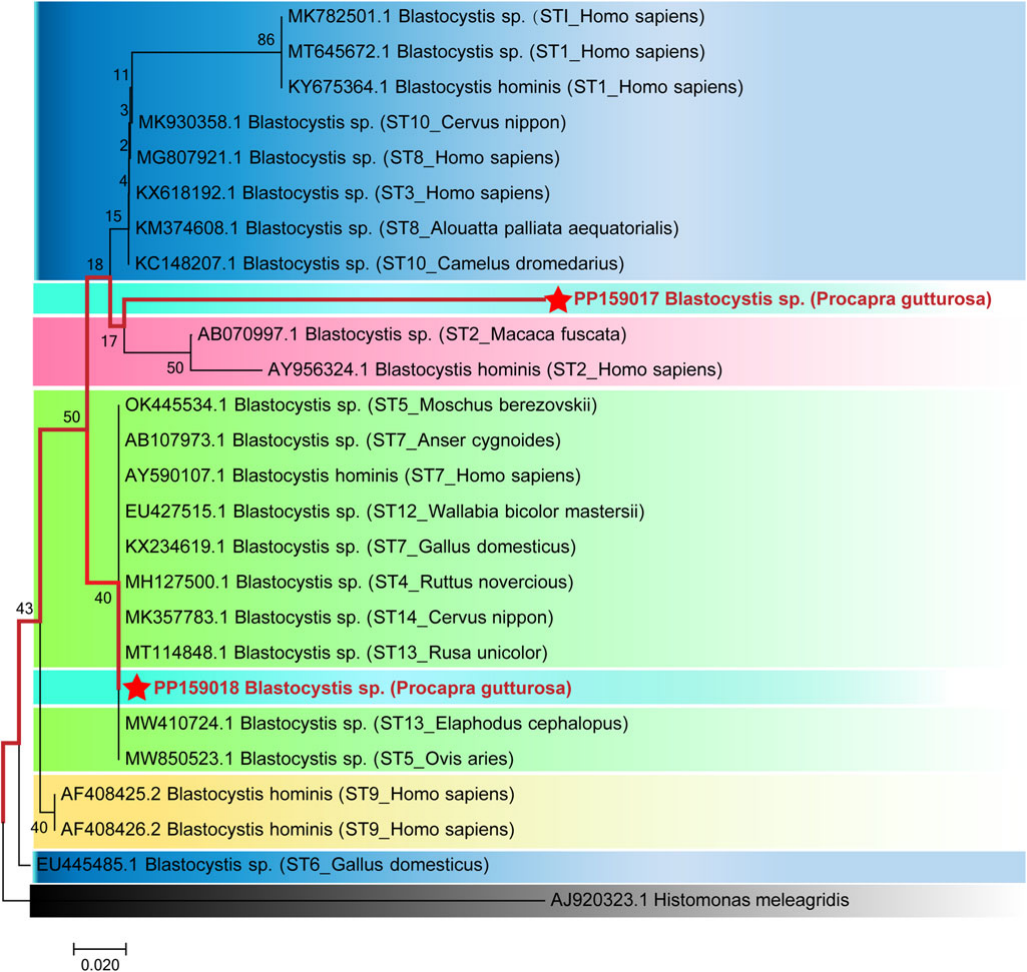
The Evolutionary relationships of *Blastocystis* sp. genotypes identified in the present study and other known genotypes and species on GenBank were inferred by a Neighbour-joining method of SSU rRNA gene using the bootstrap test (1000 replicates) and Jukes–Cantor model method. The *Histomonas meleagridis* (GenBank: AJ920323.1) were used as the outgroup. The Solid red lines and red 5-pointed stars represent novel genotypes identified in this study.

A phylogenetic tree (Fig. 3) was constructed by comparing the SSU rRNA gene sequence of *Blastocystis* sp. with the reference sequence. Based on the clustering results, the genotype of *Blastocystis* sp. (PP159017) was most closely related (88% to 89% homology) to *Rusa unicolor* (AB070997.1) ST2 serotype, clustered together with it, leading us to designate this newly discovered genotype as serotype ST2. Conversely, the genotype of *Blastocystis* sp. (PP159018) was most closely related (79% to 80% homology) with the Japanese macaque (MT114848.1) and *E. cephalopus* (MW410724.1) ST13 serotypes, and clustered with them, prompting us to assign this newly discovered genotype as serotype ST13.

### Evolutionary relationships analysis of SSU rRNA gene in *C. cayetanensis* isolated from Mongolian gazelles

**Figure 4.**
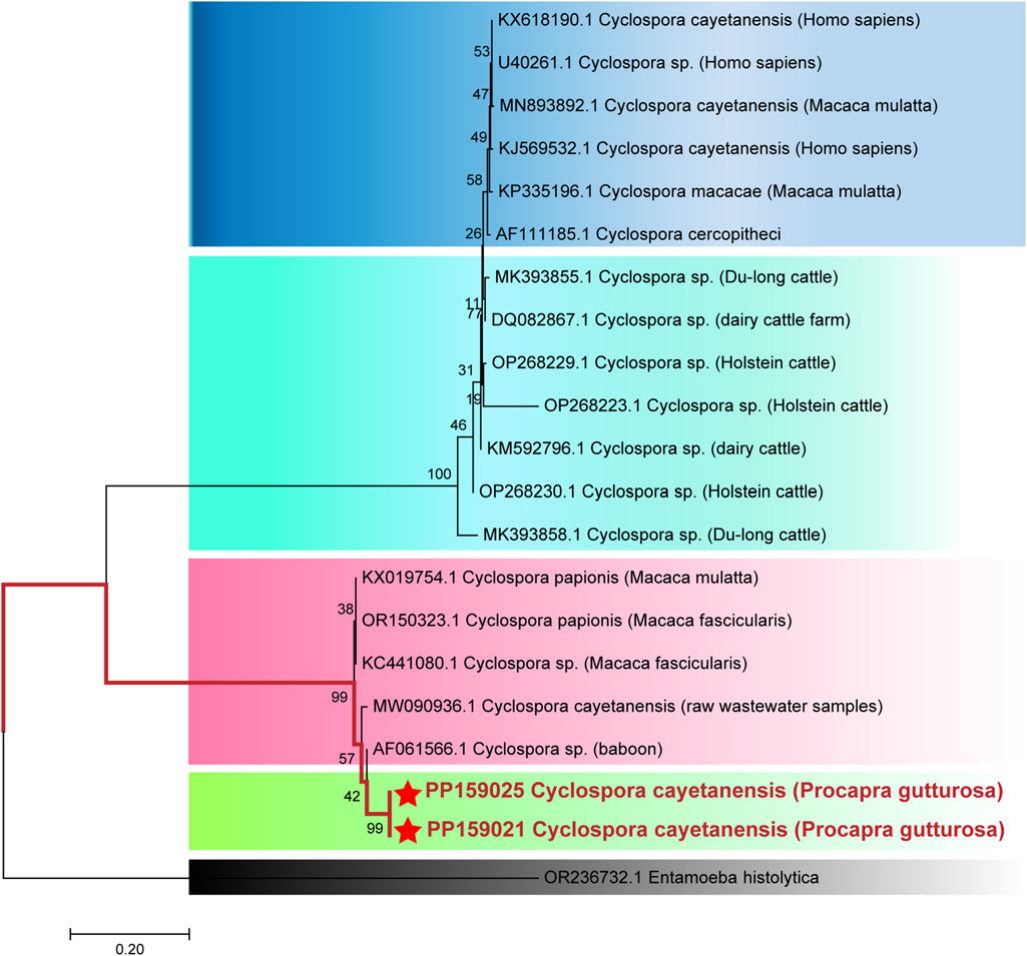
The Evolutionary relationships of *Cyclospora cayetanensis* genotypes identified in the present study and other known genotypes and species on GenBank were inferred by a Neighbour-joining method of SSU rRNA gene using the bootstrap test (1000 replicates) and Tamura 3-parameter model method. The *Entamoeba histolytica* (GenBank: OR236732.1) were used as the outgroup. The Solid red lines and red 5-pointed stars represent novel genotypes identified in this study.

A phylogenetic tree (Fig. 4) was constructed by comparing the SSU rRNA gene sequence of *C. cayetanensis* with the reference sequence. Based on the clustering results, the genotype of *C. cayetanensis* isolates from Mongolian (PP159021 and PP159025) were most closely related (94 to 95% homology) to primates (AF061566.1), with which they clustered.

## Discussion

Transboundary migratory animals play a crucial role in disease outbreaks by facilitating the transmission and spread of pathogens during their long-distance migrations. They can potentially serve as reservoirs for various novel pathogens, introducing them to new geographic areas as they traverse different regions (Uhlemann *et al*., 2017). In the case of Mongolian gazelles, many new pathogens have been discovered. For instance, an undescribed chewing louse of the genus *Damalinia* was collected from Mongolian gazelles (Lux *et al*., 1997). Furthermore, in 2014, *Taenia* spp. and *Anaplasma* were reported for the first time in Mongolian gazelles (Li *et al*., 2014). Research conducted on the nematode composition in the digestive tract of Mongolian gazelles revealed the presence of *Orloffia bisonis*, *Marshallagia mongolica*, *Nematodirus archari*, *Nematodirus andreevi*, *Trichostrongylus colubriformis* and *Trichostrongylus probolurus*. Notably, *N. archari* and *N. andreevi* were found for the first time in Mongolian gazelles, and all gastrointestinal nematode species found in Mongolian gazelles have been recorded in domestic animals within Mongolia (Kuznetsov *et al*., 2014). During migration, animals undergo physical decline, increased energy consumption, and heightened immune system stress, making them more susceptible to disease. Moreover, in comparison to other animal species, Mongolian gazelles may have more frequent close contact and interactions, potentially resulting in an increased transmission of parasites and other infectious diseases (Kuznetsov *et al*., 2014). In 1990, over 1000 Mongolian gazelles migrating from Mongolia to grazing areas in China died due to infection with Pasteurellosis (Yuan *et al*., 1991). Research conducted testing for foot-and-mouth disease virus in serum samples collected from Mongolian gazelles in eastern Mongolia and found that these viruses in Mongolian gazelles were likely due to spill-over from livestock (Nyamsuren *et al*., 2006). Studying the spillover of diseases in migratory cross-border Mongolian gazelles is of significant importance for understanding disease transmission and pathogenesis (Wang *et al*., 2009). Additionally, in the central-Mongolian border region of Inner Mongolia, there is a large population of rodents which also serve as natural hosts for parasite transmission (Feng *et al*., 2020), these rodents may have direct or indirect contact with Mongolian gazelles in geographically overlapping areas, thereby increasing the risk of disease spillover. However, there is a lack of sufficient research on the impact of cross-border migratory terrestrial animals on disease outbreaks, particularly in the case of Mongolian gazelles, when compared to migratory birds that can carry avian influenza viruses (van der Kolk, 2019). This is the reason why we did not conduct a comparative analysis and discussion of infection rates between hosts.

Most morphological differences among protozoan parasites cannot be distinguished by the naked eye, and relying solely on oocyst morphology identification has limitations. To overcome this deficiency, genetic analysis using molecular identification methods can be employed to analyse genetic variations among different species or within species of protozoan parasites (Xiao, 2010; Ryan *et al*., 2014). The 18S rRNA gene is responsible for encoding the multicopy repeat sequence of the SSU rRNA, which is approximately 1850 bp in length. It serves as a marker gene with informative and functional roles, making it the most widely used genotyping tool for identifying host-adapted genotypes of protozoan parasites (Xiao and Feng, 2017). In this study, we discovered new host-adapted genotypes of *Cryptosporidium* spp. and *C. belli* in Mongolian gazelles. Host adaptation is a common phenomenon in the genus *Cryptosporidium*, and specific genotypes are often associated with specific animal groups (Xiao *et al*., 2002). *Cryptosporidium* has been found to have various host-adapted genotypes in many studies, especially among wildlife (Hu *et al*., 2022). Studies on *C. belli* are currently mainly focused on humans, with fewer studies conducted on wildlife (Xu *et al*., 2021). The emergence of host-adapted genotypes is the result of long-term coevolutionary interactions between hosts and parasites (Vorburger and Perlman, 2018). This not only helps to shape the host immune system but also contributes to parasite genetic heterogeneity (Møller, 2008; Schwensow *et al*., 2011; Soler, 2014). Specific environmental conditions determine how any genotype of one species affects the adaptations of another species (Laine, 2009). Host-adapted genotypes are more likely to be found in the presence of co-evolving parasites if adaptation has a significant benefit to the host but little or no detrimental effect on the parasite (Ebert, 2005). Therefore, elucidating the occurrence of novel host-adapted genotypes in wildlife is of great significance for understanding host–parasite relationships, disease transmission and host evolution.

Furthermore, to date, a total of 32 genotypes (STs) of *Blastocystis* sp. have been reported based on the polymorphism within the small subunit ribosomal RNA (SSU rRNA) gene (Liu *et al*., 2022). Additionally, we identified 2 new genotypes of *Blastocystis* sp., 1 belonging to ST2 and the other belonging to ST13. Previous studies have shown that *Blastocystis* sp. ST2 is commonly found in primates, while ST13 has only been detected in wildlife so far (Cian *et al*., 2017). This indicates the presence of multiple genotypes of *Blastocystis* sp. in Mongolian gazelles, posing a risk of zoonotic infection. In addition, a total of 19 genotypes of *Cyclospora* have been reported, including those capable of infecting non-human primates (Li *et al*., 2015). Among them, *C. cayetanensis* has been confirmed as a zoonotic pathogen and is the only known genotype capable of infecting humans (Ortega and Sanchez, 2010). The clustering of the *C. cayetanensis* genotype we identified with sequences obtained from non-human primate hosts in the same branch suggests a close genetic evolutionary relationship. This suggests that Mongolian gazelles may have a connection to their contact with the human environment.

In summary, this study represents the first time to investigate the infection status of 4 zoonotic intestinal protozoan parasites (*Cryptosporidium* spp., *C. belli*, *Blastocystis* sp. and *C. cayetanensis*) in migratory Mongolian gazelles. Among these, infection with *C. cayetanensis* is higher and was the dominant species in this study. The identification of recently discovered host-adapted genotypes within this study suggests that the Mongolian gazelle population may harbour a multitude of uncharacterized or novel pathogen genotypes, increasing the likelihood of pathogen spillover. However, investigations into virulence are limited by the scarcity of parasites isolated from samples, which will be a primary focus of our subsequent research. Therefore, it is crucial to enhance active surveillance measures and conduct comprehensive investigations into reverse pathogenesis on unique wildlife species in cross-border regions.

## Data Availability

The data that support the findings of this study are available from the corresponding author upon reasonable request.
